# Everything You Always Wanted to Know about β_3_-AR * (* But Were Afraid to Ask)

**DOI:** 10.3390/cells8040357

**Published:** 2019-04-16

**Authors:** Giorgia Schena, Michael J. Caplan

**Affiliations:** Department of Cellular and Molecular Physiology, Yale University School of Medicine, New Haven, CT 06510, USA; michael.caplan@yale.edu

**Keywords:** beta-3 adrenergic receptor, therapeutic target, G-protein coupled receptors

## Abstract

The beta-3 adrenergic receptor (β_3_-AR) is by far the least studied isotype of the beta-adrenergic sub-family. Despite its study being long hampered by the lack of suitable animal and cellular models and inter-species differences, a substantial body of literature on the subject has built up in the last three decades and the physiology of β_3_-AR is unraveling quickly. As will become evident in this work, β_3_-AR is emerging as an appealing target for novel pharmacological approaches in several clinical areas involving metabolic, cardiovascular, urinary, and ocular disease. In this review, we will discuss the most recent advances regarding β_3_-AR signaling and function and summarize how these findings translate, or may do so, into current clinical practice highlighting β_3_-AR’s great potential as a novel therapeutic target in a wide range of human conditions.

## 1. Introduction

Soon after the subdivision of β-adrenoceptors into subtypes β_1_ and β_2_, it became clear that the pharmacological profile of some apparently β-adrenoceptor-mediated responses did not fit either of these two subtypes [1]. However, the existence of a third subtype, β_3_-AR, was not universally accepted until it was first cloned in 1989 [2]. Ever since then, β_3_-AR expression has been reported in several tissues revealing, completely or in part, new details on its signaling pathways and on the variety of functions that it mediates. New aspects of β_3_-AR have recently been described, involving it in urine concentrating mechanisms [3], fat mass reduction [4], and inflammatory processes [5,6] thus opening the way for new potential therapeutic applications. Importantly, β_3_-AR agonist mirabegron is the first of this class of compounds that has been approved for the treatment of overactive bladder syndrome and one of the few alternatives to anticholinergic medications [7]. Given β_3_-AR expression and signaling in districts such as myocardium and adipose tissue, mirabegron is also being tested in repurposing trials for cardiovascular and metabolic conditions (commented on in [8,9]). Consistently, β_3_-AR’s most common genetic variant, Trp64Arg, has been found to positively correlate with metabolic parameters such as HDL-C (High Density Lipoprotein-Cholesterol) and LDL-C (Low Density Lipoprotein-Cholesterol) accumulation rates and glucose tolerance [10,11]. After a brief overview of basic information regarding gene structure, protein, and pharmacology of the receptor, we will focus on β_3_-AR role in some of the main tissues where its expression has been reported. In each subsection, we will review the state of the art in signaling pathways and potential therapeutic approaches with a focus on studies in humans and rodents.

## 2. Gene and Protein

The gene encoding β_3_-AR has been identified in several species such as rat [12], mouse [13], bovine, sheep, goat [14], and dog [15]. In humans, the gene is localized on chromosome 8 and shares a 51% and 46% identity with β_1_- and β_2_-AR amino-acid sequences, respectively. This is mostly limited to the transmembrane domains and membrane-proximal regions of the intracellular loops, parts of the receptor respectively involved in ligand binding and G-protein interactions [16,17]. The mouse and human β_3_-AR are 81% identical, with the highest homology in the transmembrane domains (94%), and the lowest in the C-terminal tail and third intracellular loop. Consistently, their C-terminal regions differ in sequence and length, ranging from 6 (human) to 12 (mouse and rat) additional residues. β_3_-AR gene also contains introns. The number of exons/introns of the human and rodent genes differs and can be summarized as follows: the human β_3_-AR gene is composed of two exons and a single intron while in mouse, there are three exons and two introns [18]. In both species, exon 1 spans the 5′ untranslated region and the major part of the coding block; however, in humans, exon 2 only contains 19 bp of the coding region (last 6 aa of the C-terminus) and the 3‘ untranslated region; in mouse it contains 37 coding nucleotides and 31 bp of 3‘ untranslated region, the remainder of which is carried by exon 3 [19]. Despite intron presence, no spliced variants have been described for the human β_3_-AR gene, whereas two have been characterized in the mouse [19,20,21].

β_3_-AR gene encodes a single 408–amino acid residue–long peptide chain that, together with β_1_- and β_2_-AR, belongs to the G-protein coupled receptor (GPCR) family characterized by seven transmembrane (TM) domains, with three intracellular and three extracellular loops. The N-terminal region of β_3_-AR is extracellular and glycosylated, whereas the C-terminus is intracellular. Unlike in β_1_- and β_2_-AR, it lacks sites for phosphorylation by protein kinase A (PKA) and β-adrenoceptor kinase (βARK) thereby making β_3_-AR relatively resistant to desensitization [17,22,23]. Among others, this feature contributed in making β_3_-AR an interesting therapeutic target that would potentially be suitable for chronic treatments. From a molecular point of view, desensitization of β_3_-AR might not be mediated by internalization/degradation of the receptor, as for β_1_-AR [24], but rather by downregulation of downstream components of the signaling pathway [25] or even mRNA regulation (reviewed in [23]). However, caution must be used when evaluating the data present in literature as results were not always consistent (reviewed in [23]). Transmembrane domains TM3, TM4, TM5, and TM6 are essential for ligand binding, whereas TM2 and TM7 are involved in Gαs activation [26]. The disulphide bond between extracellular loops 2 and 3 is also essential for ligand binding and activity of the receptor. The Cys361 residue in the fourth intracellular loop is palmitoylated. Palmitoylation has been shown to mediate adenylyl cyclase stimulation by the agonist bound receptor, possibly by promoting the insertion of several adjacent residues into the membrane and thus forming an additional intracellular loop, resulting in an active conformation for G-protein coupling [27].

## 3. Pharmacology of the Receptor

The criteria that are used to define a characteristic β_3_-AR pharmacological response have been defined in several studies [26,28,29,30,31,32] and can be summarized as follows. β_3_-AR has high affinity and potency for selective agonists such as mirabegron, vibegron, solabegron, and ritobegron; partial agonist activity of β_1_- and β_2_-AR antagonists, such as CGP12177A, bucindolol, and pindolol; an atypically low affinity for β-AR antagonists such as propranolol and nadolol; and lastly, poor stereoselectivity for reference agonist and antagonist enantiomers in respect to the values reported for traditional β_1_- and β_2_-AR. Several studies that clarify β_3_-AR pharmacology were published in the last 20 years and are summarized in a comprehensive review by Donny Strosberg [33]. Structurally, most of the ligands share a similar backbone, with three domains: a left- and right-hand side connected by a linker. The left-hand side is typically an arylethanolamine or aryloxypropanolamine [34], the linker has various structures including both aromatic and aliphatic moieties, the right-hand side typically contains polar and/or ionizable functionalities [35]. A great number of ligands have been tested so far, but their assignment to either one or the other category remains controversial [36,37].

### 3.1. Agonists

β_3_-AR agonists fall in two classes depending on the time of their discovery [35]: the first-generation compounds such as BRL37344 and CL316243, were developed in the 1990s while the second-generation followed or were improved later (see Table 1 for a list of agonists tested in clinical trials).

BRL37344 is possibly the most widely used of all of the β_3_-AR agonists in both human and mouse settings, but its pharmacology is controversial. It displays a higher affinity for rodent β_3_-AR vs. human β_3_-AR and also higher potency measured as the ability in stimulating adenylyl cyclase [13,48]. Nonetheless, BRL37344 was recently used to corroborate the hypothesis that β_3_-ARs are involved in the relaxation of human detrusor muscle and of human corpus cavernosum [49,50]. More than one study has reported high potency (pEC50 = 6.5–7.5) similar to that of the non-selective agonist isoproterenol and to the selective agonist mirabegron on human bladder strips [49,51]. Finally, while some studies report that BRL37344 has a higher selectivity for β_3_-AR vs. β_1_- and β_2_-AR [31] some others point out that it is able to activate other β-ARs [52,53]. CL316243 exhibits a low potency but a good selectivity for the human β_3_-AR: >128- and 10-fold higher than β_1_- and β_2_-AR, respectively [28]. It has been used in studies on the relaxation of pre-contracted human detrusor [54]. CL316243 appears to be more selective against the rodent β_3_-AR, as its potency to stimulate cAMP formation in transfected CHO cells was found to be higher with mouse vs. human β_3_-AR (pEC50 8.7 vs. 4.3) [55]. CL316243 was recently found to elicit significant anabolic effects in mouse skeletal muscle [56] although the authors do not address the possibility of a simultaneous activation of β_2_-AR, the predominant isotype in skeletal muscle [57]. It was also used in a study addressing potential therapies for the vascular complication of diabetes, where it reduced the levels of reactive oxygen species and improved endothelium-dependent relaxation [58].

Second-generation agonists include FDA-approved molecules like mirabegron (YM178), yet discontinued ones such as amibegron (SR58611A) and other molecules that are currently being validated in phase II and III clinical trials, such as solabegron (GW427353) and ritobegron (KUC-7483). Mirabegron, solabegron, and KUC-7322 (the active metabolite of ritobegron) have been tested for their abilities to induce human detrusor relaxation. Mirabegron and solabegron were proven to be highly selective in stimulating cAMP accumulation in CHO cells transfected with human β_3_-AR (EC50 ≈ 22 nM), with intrinsic activities comparable to reference agonist isoproterenol. When tested on pre-contracted human bladder strips, mirabegron produced a concentration-dependent relaxation with an EC50 of 0.78 μM, similar to isoproterenol (EC50 = 0.28 μM) [38] while ritobegron had an EC50 of 1.1 μM [44]. Solabegron has been found to relax isolated human bladder strips at low concentrations and this effect was partially counteracted by pre-incubation with selective antagonist SR59230A [59]. It also reduced detrusor spontaneous contractile activity by almost 80%. Amibegron was first described for its inhibitory effect on rat colon motility [39]. Subsequently, Simiand et al. showed its antidepressant properties in predictive mechanistic models [41], but these studies were halted due to the lack of proof of β_3_-AR expression in the central nervous system. Finally, when β_3_-AR presence was reported in humans and rodents [60,61], several studies described the potential use of amibegron as an antidepressant and antianxiolytic drug in rodents, reporting good efficacy, selectivity for β_3_-AR, and overall lack of side effects [62,63,64,65].

With β_3_-AR gaining more and more importance as a therapeutic target, the screening for new compounds is progressing at a fast pace [66,67]. Of note, an innovative approach integrating energetic analysis, structure-based pharmacophores, and virtual screening was successfully used to screen 233,450 compounds for active β_3_-AR agonists and to identify common structural characteristics in the active molecules [68].

### 3.2. Antagonists

A key reason that β_3_-AR was identified later than β_1_- and β_2_-AR is its much lower affinity for classical β-AR antagonists. For instance, propranolol and bupranolol showed 140- and 17-fold higher affinity for β_1_- and β_2_-AR and >500- and 30-fold higher affinity for β_1_- and β_2_-AR, respectively [28,31]. Two of the most widely used β_3_-AR antagonists are SR59230A and L748337. SR59230A belongs to the class of aryloxypropanolaminotetralins [69] and was first listed as a putative β_3_-AR antagonist in rat gut while also displaying interesting properties in rat Brown Adipose Tissue (BAT) blunting β_3_-AR-mediated cAMP increase [70]. Despite its wide use in antagonizing β_3_-AR-mediated responses [71,72,73], SR59230A has been reported to be essentially non-selective and if anything to have a lower affinity for human β_3_-AR vs. β_1_- and β_2_-AR [31,37,51,74]. It also displayed a partial agonistic activity, promoting cAMP accumulation in CHO cells transfected with the mouse β_3_-AR receptor [75] as well as increases in the extracellular acidification rate (ECAR) [76]. Lastly, this compound also behaves as a strong biased agonist for other signaling pathways involving β_3_-AR [76]. These findings limit the use of SR59230A as an antagonist to identify β_3_-AR responses, at least when not assessed in combination with other compounds.

Pre-incubation with L748337 blunted cAMP accumulation in CHO cells expressing human β_3_-AR and also reduced glycerol production in rhesus monkey adipose tissue. It was reported to have a 100-fold higher affinity (Ki = 4 nM) for the human β_3_-AR vs. β_1_- and β_2_-AR, and also vs. the rat β_3_-AR [77]. Accordingly, a tritiated version of L748337 with a 10-times higher affinity for the human receptor against the rodent was proposed as a β_3_-selective radioligand [78]. As seen for SR59230A, it was reported to have a significant agonistic activity in promoting phosphorylation of extracellular signal regulated kinase 1/2 (Erk1/2) with a pEC50 of 11.6 nM, and also in increasing ECAR with efficacy similar to that of reference agonist, zinterol [79]. The low affinity for rat β_3_-AR might render L748337 not fully suitable for experimental studies on the receptor [78]. Furthermore, the difference in its affinity for the human and rat receptors suggests that it may be useful in mapping the receptor’s antagonist binding sites and exploring its structure–function relationships. Thus, L748337 is currently one of the very few antagonists with good selectivity for human β_3_-AR on the market.

## 4. β_3_-AR as Therapeutic Target

**Figure 1:**
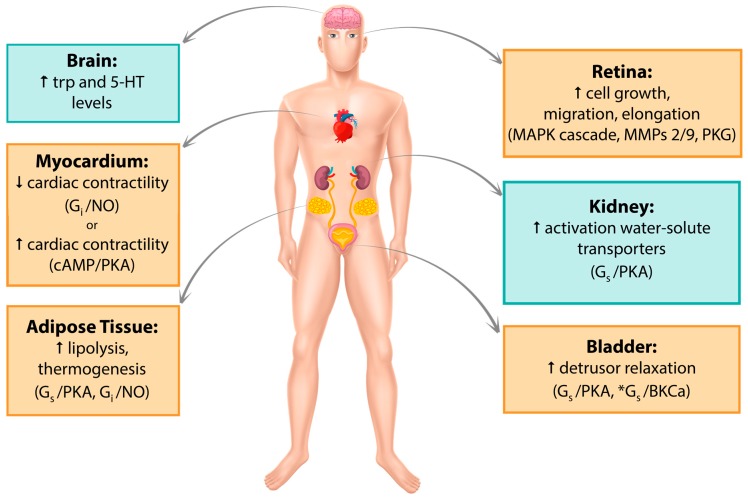
Graphic summary of β_3_-AR localization. Pathways that have been studied and reported in human tissue/cell lines are in yellow boxes. Pathways studied in mouse but not yet found in humans are in blue boxes. *Alternative cAMP-independent pathway. Trp, tryptophan; 5-HT, 5-hydroxytryptamine; MAPK, mitogen-activated protein kinase; MMPs, metalloproteinases, PKG, protein kinase G; NO, nitric oxide; PKA, protein kinase A.

Since its discovery in the late 1980s, β_3_-AR has been detected in several human tissues such as myocardium, retina, myometrium, adipose tissue, gallbladder, brain, urinary bladder, and blood vessels. Its expression has been recorded both at the mRNA and protein levels, and its activation involves a variety of cellular pathways. A recent comprehensive quantitative analysis of the human transcriptome reported β_3_-AR expression to be far more restricted than previously hypothesized [80]; however, significant findings continue to be published. In the following sections, major tissues in which β_3_-AR expression has been studied so far, their associated signaling pathways and clinical implications in humans will be discussed. Table 2 and Figure 1 summarize the data reported in this section.

### 4.1. Urinary System

β_3_-AR has been localized in kidneys [3] and in the lower urinary tract [81] including ureters [82], urethra [83], prostate [84], and bladder [85] where it appears to be the predominant subtype [86] although this idea was recently challenged in a wide transcriptomics analysis across several human tissues [80].

The presence of an atypical β-receptor in human detrusor was first suggested in the 1970s [1,87] and was confirmed by evidence that muscle relaxation in pre-contracted samples could not be evoked by stimulation with β_1_- and β_2_-AR agonists but only with β_3_-AR agonists such as BRL37344A. Consistently, β_3_-AR antagonists SR58894A and L748337 both induced a rightward shift in the concentration–relaxation curve of isoproterenol-treated detrusor muscle preparations [54,88]. The presence of β_3_-AR transcript was first reported in human detrusor smooth muscle cells [88] while the protein was found later on in the detrusor and urothelium [85]. Determination of the relative abundance of each one of the three β-AR in the bladder muscle by quantitative analysis revealed β_3_-AR mRNA to be the most represented (97%) against 1.5% and 1.4% of β_1_- and β_2_-AR respectively [86]. However, the physiological significance of this result is limited as the tissue mRNA concentration does not necessarily correlate with the protein level.

As for the β_3_-AR signaling pathway in the bladder, several different and not always consistent hypotheses have been offered. Beta adrenergic signaling often relies on the cAMP/PKA pathway [89,90,91] and cAMP is a key player in smooth muscle relaxation, however the extent of its involvement in this process is still a matter of debate. Stimulation of non-contracted muscle strips with the β_3_-AR agonist FR165101 have been shown to induce a concentration dependent decrease in muscle basal tension and an increase in the cAMP levels. Furthermore, pre-incubation with adenylate cyclase inhibitor SQ22536 markedly suppressed both of these effects [92]. However, Maki et al. recently reported a milder effect of SQ22536 on human and pig pre-contracted detrusor strips treated with mirabegron supporting the hypothesis of a cAMP-independent mechanism, potentially via activation of myosin light chain phosphatase [93]. Along the same line, Frazier et al. previously showed that only Rp-cAMPS, among other PKA inhibitors had a small significant effect on isoproterenol responses against passive tension on non-contracted rat bladder strips, not supporting a major role for cAMP and/or PKA in β-AR-mediated bladder relaxation. They hypothesized an alternate signaling pathway involving a direct interaction of β-AR, or its linked Gs, with a different partner such as the large conductance Ca^2+^ activated K^+^ channel (BKCa) [94]. Both Uchida and Frazier showed that BKCa selective block had a negative effect on isoproterenol-stimulated pre-contracted rat bladder strips. In conclusion, these studies support a potential, and somewhat partial role, for cAMP/PKA pathway in β-adrenergic-mediated relaxation from passive tension of rat detrusor muscle; at the same time, they hypothesize a cAMP-independent mechanism involving BKCa channels in β-adrenergic modulation of smooth muscle active tone in the urinary bladder. Thus, the contribution of the two mechanisms seems to be dependent on the condition of the detrusor muscle. Additionally, D’Agostino et al. used electrical field stimulation (EFS) to show that β_3_-AR activation strikingly inhibits EFS-evoked contraction and acetylcholine (Ach) release from cholinergic nerves in a concentration-dependent manner in human detrusor bladder strips [45]. The fact that BKCa channel activation at prejunctional sites has a hyperpolarizing effect on parasympathetic nerve terminals [95] and that they are potentially involved in β_3_-AR signaling [92,94] might explain how β_3_-AR stimulation reduces neurotransmitter release. β_3_-AR modulation of ACh release is also supported by a recent immunohistochemistry study showing β_3_-AR expression on the detrusor cholinergic fibers in close proximity to sympathetic bundles innervating the human bladder. This arrangement is physiologically logical, as adrenergic fibers can release noradrenalin necessary to activate β_3_-AR [96].

Scarce and controversial information regarding β_3_-AR presence in the kidney has been reported up to date, with some publications describing functional and transcriptional evidence [97,98,99] while some others fail to report its presence at all [80]. Recently, we provided evidence regarding the localization of β_3_-AR to the vasopressin-sensitive segments of the mouse nephron. The agonist BRL37344 was used on freshly isolated kidney tubules and slices from wt and β_3_-AR knock out (KO) mice to demonstrate β_3_-AR coupling to Gs/adenylyl cyclase and its positive effects on key water and solute transporters, such as Aquaporin 2 (AQP2) and Na-K-2Cl (NKCC2). All effects reported were blocked upon pre-incubation with the antagonist L748337 or PKA inhibitor H89, suggesting a potential activation of the cAMP/PKA axis. Ex vivo findings are strengthened by in vivo data, showing that KO mice for β_3_-AR are mildly polyuric and highlighting a role for β_3_-AR in renal homeostasis. Interestingly, administration of BRL37344 to a mouse model of X-linked nephrogenic diabetes insipidus (NDI), known to be heavily polyuric, completely reverted the phenotype within 1 h from injection [3]. In light of these findings, it is tempting to speculate on the use of β_3_-AR as therapeutic target in conditions where water-solute homeostasis and/or vasopressin signaling are impaired (see Section 6).

β_3_-AR are also present in the lower urinary tract where they undergo pathophysiological changes in expression upon the initiation of lower urinary tract symptoms due to ureteral stenosis [82], chronic kidney disease, end stage renal disease, or certain carcinomas [100]. The β_3_-AR agonist mirabegron for the oral pharmacotherapy of patients affected by overactive bladder syndrome (OAB) is currently the most promising agent in over 30 years. This drug has been extensively studied, with a good number of Phase II and Phase III trials all over the world, exhibiting a good balance between efficacy and tolerability in all of them [7]. It was licensed for the treatment of OAB and approved for use in Japan in 2011 (Betanis), USA and Canada in 2012 (Myrbetriq), and Europe in 2013 (Betmiga). Safety and tolerability of mirabegron in the treatment of OAB have been extensively reviewed by Michel et al. [101]. Several repurposing studies are currently exploring potential uses for mirabegron in the treatment of heart and metabolic conditions. Other molecules have made it to Phase III clinical trials: ritobegron however appears to have been discontinued (Clinicaltrials.gov, NCT01003405) while the new potent and selective β_3_-AR agonist, vibegron, has recently passed the later stages [47] and has been approved in Japan for the treatment of OAB [46].

### 4.2. Adipose Tissue

β_3_-AR is abundantly expressed in rodent white (WAT) and brown (BAT) adipose tissue where it mediates lipolysis and thermogenesis [13,102]. In humans, β_3_-AR mRNA levels appeared to be much lower in these tissues. The transcript was mainly identified in infant peri-renal BAT and in various deposits of WAT in the adults [18,103] while its presence at the protein level was published later through the use of a monoclonal antibody directed against the human receptor [104,105]. Of note, one of the greatest obstacles in the study of β_3_-AR role and function in humans is represented by the little presence of BAT in adults; however, studies in the last decade are overturning these findings, showing proof for a metabolic role of BAT in healthy adults [106,107]. Similarly, to rodents, selective activation of β_3_-AR stimulates lipolysis in human isolated white adipocytes [108,109].

A major trigger of both lipolysis and thermogenesis is the sympathetic nervous system. From a molecular point of view, noradrenaline (NA) released by sympathetic nerve endings binds to β_3_-AR, which couples to the α-subunit of Gs-proteins and triggers the activation of the cAMP-PKA axis. Final targets of this cascade include lipid droplet proteins, like hormone-sensitive lipase (HSL) and perilipin, that start the lipolytic process in white adipocytes. However, β_3_-AR signaling in adipocytes is promiscuous in that HSL activation can also be triggered by β_3_-AR coupling to Gi and consequent initiation of the ERK1/2 MAP kinase cascade [110,111]. The net result is the release of free fatty acids (FFAs) derived from the hydrolysis of triglycerides stored in the lipid droplet. In brown adipocytes FFAs activate Uncoupling-Protein 1 (UCP1) in the mitochondria, triggering thermogenesis. Of note, β_3_-AR activation can also lead to lipolysis by increasing the transcription/expression of the inducible nitric oxide synthase (iNOS) and thus nitric oxide levels in a PKA-dependent fashion [112].

β_3_-AR activation was recently found to be involved in the generation of hyperthermia arising from WAT, instead of the more classically thermogenic BAT, with simultaneous WAT remodeling and increased beiging in mice with impairment in triglyceride storage capabilities [5]. β_3_-AR KO mice also showed impaired cold-induced thermogenesis with a reduction in white adipocyte beiging [113]. This is consistent with recent findings that β_3_-AR activation induces trans-differentiation of mature white adipocytes but that cold-induced beiging occurs via β_1_-AR [114]. In contrast, others argued that β_3_-AR is dispensable for thermogenesis, showing that adaptive response to cold is not affected in KO mice [115].

Regardless of these effects, β_3_-AR KO mice lack a major phenotype, with only moderate fat accumulation in females vs. males and no altered adipose-related functional response to β-agonists [116]. KO mice also show a compensatory over-expression of β_1_-AR in WAT and BAT and they are able to survive cold exposure through β_1_-AR mediated thermogenesis and UCP1 increase [116,117]. More recent findings, however, reported that β_3_-AR KO mice generated on the same background show an increased susceptibility to high fat diet, developing more severe obesity with white adipocyte hypertrophy and inflammation in comparison to wild type mice [118]. Consistently, Hong at al showed that the ERK-β_3_-AR axis is a key player in obesity-driven increases in lipolysis and that administration of MEK inhibitors is efficient in blunting both in vivo lipolysis in diet-induced obese mice and ex vivo lipolysis induced by β_3_-AR agonist treatment on human and murine WAT explants [119].

Lipid and carbohydrate metabolism are influenced by β_3_-AR agonists with normalizing effects on hyperinsulinemia, increases in resting energy expenditure (REE) and decreases in circulating FFAs, fat/non-fat mass ratio and body weight gain in rats or in obese mice [4,120,121]. In humans, β_3_-AR expression level is reduced in obese patients [105]. These observations raised interest in the development of compounds for the treatment of obesity and type 2 diabetes [122,123], but clinical studies [124,125] were disappointing due to poor selectivity of the drugs for the human β_3_-AR and different contributions of white and brown adipocytes in rodents and humans [126].

No agonist has yet progressed beyond phase II clinical trials. It is unclear whether this is because of the lack of compounds with suitable drug properties (e.g., oral bioavailability), because of a lesser role for β_3_-AR in modulating adipogenesis in humans than in rodents or due to the little presence of BAT in adults, although this concept has been challenged in the last decade [106,107]. However, there is still active speculation in regard to β_3_-AR as a target for metabolic conditions. A short review by Jonathan Arch. discusses this long-standing issue, proposing some alternatives like combined administration of β_3_-AR agonists and other compounds aimed at increasing BAT amount in patients [127]. A recent study investigated the repurposing of mirabegron, determining its ability to stimulate BAT activity and REE in healthy subjects [9]. Despite study limitations of size and gender, these findings show that administration of supra-therapeutic doses of mirabegron leads to an increase in REE via cardiovascular stimulation and BAT activation but also causes unwanted cardiovascular effects, likely due to β_1_-AR off-target binding. Finally, even in cases where weight reduction is not achieved, β_3_-AR stimulation could generally exert positive metabolic effects [9]. This study might contribute to the re-opening of a debate about β_3_-AR role as a possible candidate for the treatment of obesity and metabolic diseases, highlighting the necessity for the development of more subtype-selective agonists so as to limit side effects to a minimum.

Along the same line and based on all these pre-clinical and clinical considerations, new beta-phenylethylamine compounds were recently synthetized and tested in rats with alloxan-induced type II diabetes. All of these compounds markedly decreased levels of total cholesterol, LDL cholesterol, and triglycerides and increased the values of antiatherogenic HDL cholesterol. Moreover, the effects were significantly more intense than the reference substance BRL37344 [128]. In light of these considerations it appears that, although theoretically appealing, the amount of data that is currently available is not enough for β_3_-AR agonists to proceed into a clinical stage and more insights into the receptor physiology are ultimately needed for this purpose.

### 4.3. Myocardium

The presence of β_3_-AR transcript [103,129] and protein [104,105] in cardiac tissue was described in the early 1990s in both atria and ventricles. However, reports varied significantly depending on the portion of cardiac tissue considered with some failing to identify any transcript in ventricles at all [130]. Significant inter-species differences have also been observed in response to β_3_-AR activation [131]. In line with the purpose of this work, only effects in humans and rodents will be reviewed in this section. In human ventricles, β_3_-AR was shown to exert a marked negative inotropic effect upon stimulation with agonists such as BRL37344, amibegron, and CL316243. However, these compounds were shown later on to exert non-specific effects through β_1_- and β_2_-AR [132]. Since this effect was attenuated by pre-treatment with the pertussis toxin, it was attributed to a potential coupling with Gi proteins through an as yet unknown mechanism [129]. Among the pathways involved in cardiac contractility, the nitric oxide (NO) pathway plays a role in decreasing cardiac contraction upon activation of the autonomic nervous system. Indeed, the effect of β_3_-AR stimulation on cardiac contractility is abrogated by pre-incubation with either a non-specific NO blocker or a nitric oxide synthase (NOS) inhibitor and treatment with BRL37344 induces an increase in both NO and cGMP, known mediators of the NO pathway. The NOS isoform most likely involved is the endothelial NOS (eNOS), abundantly expressed in human cardiac myocytes [133]. The effect of NO, endogenously-produced by eNOS on cardiac contractility, in human myocytes is consistent with data obtained in other species [134,135,136]. However, β_3_-AR can also modulate NO signaling via neuronal NOS (nNOS) [137,138]. Interestingly, the signaling through the downstream Gi/NOS pathway involves increases in intracellular cGMP, which are sensitive to NOS inhibitors and protein kinase G (PKG) activation [139]. Functional evidence for a NO-cGMP-PKG pathway leading to ventricular relaxation in rats has been discussed by Angelone et al. that showed how treatment with hemoglobin (a NO scavenger), l-NMMA (a NOS inhibitor), or ODQ (a soluble guanylate inhibitor) abolished the effect of BRL37344 [140]. Unexpectedly, another study showed a positive inotropic effect and a stimulation of L-type Ca2^+^ channel-mediated current (*I*_Ca-L_) in isolated human atrial myocytes upon β_3_-AR stimulation with BRL37344 and partial agonist CGP12177. These effects were neither due to Gi nor to NOS activation but were instead mediated by a cAMP/PKA pathway, as pre-incubation with the selective PKA inhibitor H89 abolished them completely [141]. However, according to Christ et al., the BRL37344-mediated increase in contractility elicited in human right atrial appendages is attributable to β_1_- and β_2_-AR stimulation. This is possible because BRL37344 has been reported to induce preferential activation of β_2_-AR in human atria and its effect is only mildly antagonized by L748337 while it is heavily blunted by β_2_-AR (and β_1_-AR) antagonists [142]. Some other studies completely failed to show any inotropic effect in human ventricular muscle samples although confirming a positive coupling between β_3_-AR stimulation and *I*_Ca-L_ in both atrial and ventricular chambers [143]. Of note, these data have to be interpreted carefully taking into account the poorly defined pharmacology of the receptor, which may give rise to selectivity problems between isotypes. Additionally, a major limitation of these studies often lies in fact the agonist-induced effects are not corroborated by antagonist stimulations and/or achieved with the administration of a single concentration of drug. Indeed, the negative inotropic effect elicited by low concentrations of CL316243 and amibegron in human ventricles [129], vanishes at higher concentrations [132]. A thorough review of β_3_-AR role in the human myocardium and all the hidden dangers in the interpretation of its pharmacology has been recently published by Arioglu-Inan and colleagues [144]. If on the one hand, β_3_-AR partial responses in the myocardium might suggest that the therapeutic use of β_3_-AR agonists does not pose a risk to cardiac function; on the other, Mo et al. recently reported that mirabegron exerts both a cardiostimulant and a cardiodepressant effect seemingly unrelated to β_3_-AR activation. In this study, stimulation of human right atrial appendages with mirabegron caused an increase in contractile force that was not only antagonized, but even reversed to negative inotropy upon incubation with β_1_-AR blocker CGP20712A. However, neither effect was influenced by treatment with β_3_-AR antagonist L748337. Besides discrediting a role for β_3_-AR in mediating cardiac inotropy the authors also point out that, given mirabegron structure, a direct interaction with β_1_-AR is rather unlikely in favor of a more indirect action as a sympathomimetic drug. Indeed, preincubation of the samples with neuronal uptake blockers induces the same negative inotropic effect achieved with CGP20712A [145]. It is, indeed, of the utmost importance that such studies are taken into account during the optimization and design of future β_3_-AR agonists intended for clinical use.

The β_3_-AR signaling pathway in human myocardium can thus be summarized as follows: its stimulation leads to the activation of eNOS or nNOS, probably by means of Gi protein, increasing NO production; NO, in turn, activates a soluble form of guanylate cyclase leading to cGMP production followed by PKG activation that ultimately enhances myocyte relaxation and causes negative inotropy, possibly through the phosphorylation of troponin I, phospholamban, and L-type Ca2+ channels [146]. However, β_3_-AR downstream signaling the heart might also involve L-type Ca^2+^ channels stimulation through a cAMP/PKA mechanism leading to positive inotropy at least in human atria [141]. Finally, despite coupling with L-type Ca^2+^ channels, some studies fail to show any effect on inotropy at all [143,144]

Hence, β_3_-AR produces a negative inotropic effect in the human myocardium that is more pronounced in ventricles than in atria and opposite to the one mediated by β_1_- and β_2_-AR. Unlike the other two isotypes, β_3_-AR is expressed at a low level in the normal heart but several reports showed changes in its expression levels at both protein and transcript level in pathophysiological conditions such as diabetes, heart failure, sepsis, and myocardial fibrosis [147,148,149,150]. Due to this overexpression and its low tendency to desensitization, β_3_-AR is considered to be a promising therapeutic target for cardiac conditions. For instance, both nebivolol and metoprolol, FDA-approved β_1_-selective blockers, have been shown to exert their cardio-protective effects via β_3_-AR [151,152]. On the one hand, β_3_-AR could serve as a ‘safety valve’ at the high levels of sympathetic stimulation typical of cardiac conditions; on the other, its cardio-depressant effect might become maladaptive during later stages and lead to further myocardial dysfunctions. β_3_-AR antagonists and especially agonists have been proposed as potential strategies for treatment or prevention of heart failure and of ischemic damage [136,149]. It was shown that activation of the β_3_-AR-PKG signaling cascade inhibits hypertrophy in transgenic mice models with cardiac specific expression of the human receptor and that selective β_3_-AR stimulation confers protection in the failing heart through a specific cGMP/NO signaling pathway [153]. NO has also been associated with the enhancement of endothelial cell proliferation [154] that can have a beneficial effect on cardiac function and remodeling within the failing myocardium [155]. Recently, the same mouse model was used to show that the presence of functional β_3_-AR in the heart attenuates cardiac hypertrophy via the activation of nNOS. This effect is exerted through paracrine mediators secreted by β_3_-AR-expressing cardiac myocytes that ultimately attenuate the pro-fibrotic phenotype in cardiac fibroblast [147].

Based on all the pre-clinical evidence discussed above, two clinical trials, the “Beta 3 Agonist Treatment in Heart Failure” (Beat-HF; clinicaltrials.gov: NCT01876433) and the “Assessment of Efficacy of Mirabegron, a New Beta 3-Adrenergic Receptor in the Prevention of Heart Failure” (Beta3_LVH; clinicaltrials.gov: NCT02599480), are evaluating the effects of β_3_-AR agonism on HF progression and development (commented on in [8]).

### 4.4. Brain

Antidepressant effects mediated by a so-called atypical β-receptor in rat brain [41], but attributed by some to β_1_-AR [156], were described in the early 1990s. The expression of β_3_-AR mRNA in the rat brain [61] and in different regions of the human brain such as frontal cortex and hippocampus [60] was reported a little later. Given the fact that brain β-ARs are involved in the regulation of depressive states [157], Stemmelin et al. conducted a study on “the first selective, orally active and brain-penetrant β_3_-AR agonist”, amibegron, with high efficacy and potency at the rat and human β_3_-AR, demonstrating its robust anxiolytic-like effects in rats [64]. Indeed, treatment with amibegron increased tryptophan and consequently 5-hydroxytryptamine (5-HT) levels via activation of peripheral β_3_-AR in several areas of rat brain and also NE release via activation of central β_3_-AR probably located in the locus coeruleus, [158]. Other studies suggested the involvement of neurotrophic or anti-apoptotic factors and interactions with serotonin 5-HT1A, 5-HT2A, and 5-HT3 receptors in the β_3_-AR-mediated antidepressant effect [159,160]. Amibegron has been investigated through several clinical trials in 2007 but Sanofi has discontinued its clinical development for the time being due to lack of efficacy [40].

β_3_-AR is also densely expressed in the murine hippocampus [161] where it acts to modulate glucose uptake. It has consistently been shown to be involved in memory consolidation, a process that relies both on modulation of hippocampal neuron excitability and activation of brain glucose metabolism. Church et al. recently described a role for β_3_-AR in regulating the action potential firing of hippocampal pyramidal neurons via a cAMP-independent pathway involving hyperpolarization-activated cyclic nucleotide gated channels [162]. Gibbs et al. showed that memory formation at the time of learning is enhanced by the administration of β_3_-AR agonist CL316243 to day-old chicks, a process most likely due to an increase in glucose uptake via GLUT3 [163]. Interestingly, β_3_-AR KO mice show a decrease in mRNA level of GLUT3 transporter in the amygdala and impairments in long- and short-term memory formation [164].

Finally, β_3_-AR has also been implicated in the generation of neuropathic pain as the mediator of ATP release from dorsal root ganglia neurons upon NE stimulation [165]. Recent studies further showed that only peripheral β_3_-AR are involved in the generation of pain and neuroinflammation by eliciting an increase in the release of pro-inflammatory cytokines such as TNFα and Interleukin 1β [6]. Given these considerations and the fact that β-blockers are already widely used in several areas of clinical treatment, some studies are seeking ways to include them in pain management [166].

### 4.5. Retina

β_3_-AR also exerts vasorelaxant and antiangiogenic effects in rodent retinal blood vessels [167]. Protein expression in human retina was first detected in cultured retinal endothelial cells where targeted stimulation with BRL37344 promoted cellular growth and migration [168]. A targeted approach via selective inhibition showed that the β_3_-AR pro-proliferative effect is likely mediated by a signaling cascade involving mitogen-activated protein kinase (MAPK) pathway components rather than by the cAMP/PKA axis, while β_3_-AR induced cell migration relies instead on a Src-PI3K pathway. Interestingly, functional studies on human choroidal cells showed that β_3_-AR stimulation evoked a substantially lower proliferative response in these cells compared to retinal endothelial cells and that this is likely mediated by PKG [169]. Rodent models are currently used to investigate a potential role for β_3_-AR as a therapeutic target in retinopathies [170]. In retinal vascularization-associated diseases, hypoxia plays a large role in triggering neoangiogenesis through vascular endothelial growth factor (VEGF) release. Dal Monte et al. studied β_3_-AR regulation of VEGF production in mouse retinas, showing that β_3_-AR protein levels are increased in hypoxic retinas and that siRNA silencing of the receptor blunts hypoxia-induced VEGF increase [171]. β_3_-AR stimulation with BRL37344 significantly increased VEGF release in hypoxic retinas *vs.* normoxic and untreated hypoxic samples. Conversely, its blockade prevented VEGF increase in the same hypoxic conditions, suggesting a potential role for β-blockers in the treatment of retinal diseases. However, given the physiological role of VEGF as a growth factor, it has to be taken into account that changes in its levels could be associated with cell death as reported by Casini et al. 2014 [71]. Strikingly, non-selective inhibition of nitric oxide synthase (NOS) abolished BRL37344-induced VEGF increase while supplementation of NO bypassed the beneficial effect of β_3_-AR blockade, pinpointing NO as a key downstream effector in this pathway. Of note, expression of the inducible isoform of NOS (iNOS) is not only unaffected by β_3_-AR stimulation but even reduced upon β_1_-AR activation in human retinal endothelial cells [172], suggesting that the β_3_-AR effect in human retina could involve different NOS isoforms such as eNOS or nNOS, as in the myocardium [137,138] and that data extrapolation between human and rodents should be evaluated carefully.

### 4.6. Cancer Therapy

The presence of β_3_-AR mRNA and protein has been reported across different tumors, including vascular tumors, colon and breast cancer, and human leukaemia cells [173,174,175,176,177] and the Trp64Arg polymorphism may be associated with an increased susceptibility to some cancers (see Section 5). In particular, β_3_-AR presence is detected at very high levels in human melanoma sections [99,176]. This is not surprising considering that β-ARs have been found at many sites of tumor growth and metastasis, where they are involved in multiple cellular processes that contribute to the initiation and progression of cancer [178]. Of note, Dal Monte et al. published a study elucidating the potential role for β_3_-AR in the context of melanoma. They showed that β_3_-AR blockade increases apoptosis and reduces tumor cell proliferation and vascularization, thereby slowing down melanoma growth in treated mice [179]. Importantly, the overall levels of pro-angiogenic factors were not affected by β_3_-AR blockade, suggesting that the reduced tumor vascularization is probably due to the increase in apoptosis and that the chance of systemic side-effects is minimal. β_3_-AR was also shown to enhance the proliferative advantage of tumor cells by promoting a metabolic shift towards aerobic glycolysis, known as the Warburg effect, in melanoma stem cells. Notably this effect is reverted by SR59230A treatment [180]. Another very recent report by the same group further describes a role for β_3_-AR as immune-modulator agent in melanoma. In the study, the authors show that β_3_-AR antagonism with SR59230A and siRNA injections reduces melanoma growth in vivo. This effect is due to an increase in the number of natural killer cells (NK) and CD8 T cells (CD8) as well as their cytotoxicity, and to the abrogation of the immune-suppressive cells, the so-called myeloid-derived suppressor cells (MDSC) and regulatory T cells (Treg), in the tumor microenvironment [181]. Thus, the authors showed for the first time the involvement of β_3_-AR in immune-tolerance, reinforcing the hypothesis that its targeting could actually represent an effective therapy to overcome melanoma growth. In addition to cancer cells β_3_-AR is highly expressed in stromal, inflammatory, and vascular cells of the melanoma microenvironment where it favors melanoma aggressiveness through recruitment of stromal cell precursor to the tumor site and induction of inflammatory cytokines secretion and de novo angiogenesis [176]. A comprehensive review recapitulating the state-of-the-art of preclinical findings on β_3_-AR role in melanoma has recently been published by Dal Monte et al. [182]. Main limitations of the present studies are the ill-defined pharmacological profile of the receptor and important inter-species differences that hamper the extrapolation of animal data for human applications.

Besides the limitations described above these results may still lead the way to further explorations β_3_-AR in cancer therapy or at least in melanoma treatment.

## 5. Polymorphisms

Each β-AR is known to have some commonly recognized polymorphisms in humans (for review, see [183]). Early studies in French patients and Pima Indians reported a recurring genetic variation in the β_3_-AR gene that yields a Trp64Arg substitution at the intracellular end of TM1 [184,185]. Homozygotes for Trp64Arg exhibited increased tendency to gain weight and earlier onsets of type 2 diabetes. This mutation occurs with an approximate frequency of 8–10% in the Caucasian population, 20% in the Japanese population, and 40% in Alaskan Eskimos [186]. A correlation between Trp64Arg and type 2 diabetes, likely dependent on ethnicity, has been described in a recent meta-analysis [187]. The presence of Trp64Arg was also reported to influence metabolic parameters such as LDL-C [10] and HDL-C [11] accumulation rates and resistance to weight loss in cohorts of Brazilian and Japanese patients, respectively. Consistently, Trp64 polymorphism correlates with increases in Body Mass Index [188] and has a weak but significant influence on carriers leading to greater fat mass and percentage [189]. A stronger case was recently made in a larger patient cohort of Saudis, where Trp64Arg was linked to dyslipidemias and body weight gain [190]. β_3_-AR has a prominent role in the human bladder [86]. A meta-analysis of two case-control studies including 419 patients identified that both homozygous and heterozygous carriers of the polymorphism were associated with the occurrence of OAB [191]. However, the association between Trp64Arg and metabolic alterations is controversial, with some studies reporting no correlation between the two [192,193]. For example, Teitsma and colleagues did not detect any significant association between Trp64Arg and lower urinary tract function in a cohort of more than 1000 men [194], although Trp64Arg allele was reported to be significantly more frequent in subjects with OAB (57%) than in those without (23%) [195]. The discrepancies between these two studies could be due to inclusion criteria. Also, Trp64Arg is clinically considered to encode a hypofunctional variant of the receptor, possibly associated with a reduced bladder compliance. Hence, it is possible to argue that a functional relevance of the polymorphism would become clearer in situations when the receptor is activated, such as OAB. For this reason, a statistically significant association between Trp64Arg and OAB would be more easily detectable than one between Trp64Arg and milder lower urinary tract alterations. Finally, β_3_-AR polymorphism Trp64Arg has been recently studied in relation to urate and gout with a positive ethnicity-dependent correlation [196], and in relation to breast, endometrial, and gallbladder cancer [197,198,199] in association with an increased susceptibility.

## 6. Conclusions

The therapeutic approaches described herein are only some of the ways β_3_-AR has yet been targeted. More are still under development and need to be explored. For instance, β_3_-AR mRNA and protein expression were surprisingly reported in the female reproductive system (ovaries, placenta, and Fallopian tubes) as one of the highest hits in a recent comprehensive quantitative analysis of the human transcriptome [101]. Although the role of β_3_-AR in such tissues remains unclear, its presence and function in human near-term myometrium was previously described and specific stimulation of the receptor caused marked relaxation of myometrial contractions via a cAMP/PKA mechanism [200,201]. Such findings led to the suggestion of a potential role for β_3_-AR in the management of preterm labor and of its involvement in chronic inflammatory diseases. It is a known fact that inflammation is one of the regulators of preterm labor. Indeed, Hadi et al. recently described how β_3_-AR stimulation evokes a dose-dependent anti-oxidant effect that ultimately blunts inflammation in myometrial biopsies. The study also reports, for the first time, β_3_-AR presence and anti-oxidant properties in human macrophages obtained from healthy donors [202].

Finally, as previously mentioned, β_3_-AR can act as upstream regulator of key proteins for water and solute reabsorption. It is fair to note that the β_3_-AR signaling pathway shares a high degree of similarity with the vasopressin pathway, leading to increases in water and solute uptake that are mirrored by the activation of water and solute transporters via cAMP. In addition, β_3_-AR selective stimulation rescues AQP2 function in the absence of arginine vasopressin (AVP) action and dramatically reduces the urine volume in a mouse model of X-linked NDI [3]. Thus, this pathway may constitute a valid alternative for the treatment of conditions characterized by impairments in vasopressin signaling or vasopressin type 2 receptor (AVPR2) expression, such as NDI, polycystic kidney disease (PKD), or the syndrome of inappropriate secretion of AVP (SIADH). To further support the feasibility of a chronic treatment with β_3_-AR agonists in vivo, we recently confirmed that human β_3_-AR is resistant to desensitization in kidney epithelial cells and that it elicits long-lasting downstream effects on its targets, by comparing its desensitization rate to human AVPR2 upon stimulation with selective agonists [22].

The physiology and function of β_3_-AR is rapidly being revealed, making this receptor an interesting target for novel therapeutic approaches. Its role in humans is, however, controversial and studies have suffered from the late discovery of β_3_-AR, the consequent lack of selective detection tools, and inter-species differences. Finally, thanks to advances made over the last two decades, targeting β_3_-AR has been proposed for the treatment of several conditions, with some drugs undergoing Phase II and III clinical trials and others already on the market. Restricted tissue expression and relative low tendency to desensitization are some of β_3_-AR features that would allow for a prolonged pharmacological stimulation with low systemic off-target effects. Its low sensitivity to catecholamines also suggests its potential role in pathological conditions (e.g., heart disease), thereby making it even more appealing in such a context. The data reviewed here summarize the evidence that paved the way to the use of β_3_-AR in pharmacologic treatments and that might lead to speculations on future applications of β_3_-AR as a therapeutic target in the treatment of multiple conditions.

## Figures and Tables

**Table 1:** β_3_-AR agonists tested in clinical trials.

Compound	Manufacturer/Sponsor	Therapeutic Indications	Status	References
Mirabegron (YM178)	Astellas Pharma	Overactive bladder syndrome	FDA-approved	[38]
Amibegron (SR58611A)	Sanofi	Antidepressant, antianxiolytic	Phase II and III (2005–2008) then discontinued ^1^	[39,40,41]; Clinicaltrials.gov NCT00252330
Solabegron (GW427353)	Glaxosmithkline	Overactive bladder syndrome	Phase II (ongoing)	[42,43]; Clinicaltrials.gov NCT03475706
Ritobegron (KUC-7483)	Kissei Pharmaceuticals Co. Ltd.	Overactive bladder syndrome	Phase III Discontinued ^1^	[42,43,44,45]; Clinicaltrials.gov NCT01003405
Vibegron ^2^	Kyorin Pharmaceutical Co., Ltd. and Kissei Pharmaceutical Co., Ltd.	Overactive bladder syndrome	Approved (Japan)	[46,47]

^1^ Lack of efficiency in patients. ^2^ For further reference, see Section 4.1.

**Table 2:** Main tissues of β_3_-AR expression with relative downstream signaling and current uses in clinical therapy.

Tissue		Protein	mRNA	Function	Downstream Signaling (Mediators)	Potential Therapeutic Indications	Agonists Currently Developing	Status
Urinary System	Bladder	Yes	Yes	Bladder relaxation	G_s_ (cAMP/PKA)	Treatment of Overactive bladder syndrome	Mirabegron Solabegron Vibegron	Approved Phase II Phase III
	Kidney	Yes	Yes	Water and solute reabsorption	G_s_ (cAMP/PKA)	-	-	-
Central Nervous System	Brain	No	Yes	Increase of trp and 5-HT levels	-	Antidepressants	Amibegron (SR58611A)	Discontinued
	Retina	Yes	Yes	Endothelial cell growth, migration, and elongation	MAPK cascade, metalloproteinases 2/9, PKG	-	-	-
Adipose tissue		Yes	Yes	Lipolysis, thermogenesis	G_s_ (cAMP/PKA) and G_i_ (NO)	Anti-obesity, anti-diabetic	Beta-phenylethylamine	Pre-clinical
Myocardium		Yes	Yes	Lowering/Increasing of cardiac contractility	G_i_ (NO)/ G_s_ (cAMP/PKA)	Treatment of heart failure	Mirabegron (repurposing study)	Phase II
Myometrium		Yes	Yes	Relaxation of myometrial contractions	cAMP	Management of preterm labor	-	-
